# Supplementary figures and images for: Insights into tuberculosis burden in Karachi, Pakistan: A concurrent adult tuberculosis prevalence and child Mycobacterium tuberculosis infection survey
Source: PLOS Glob Public Health. 2024 Aug 28;4(8):e0002155. doi: 10.1371/journal.pgph.0002155 (PMC11356439; doi:10.1371/journal.pgph.0002155)

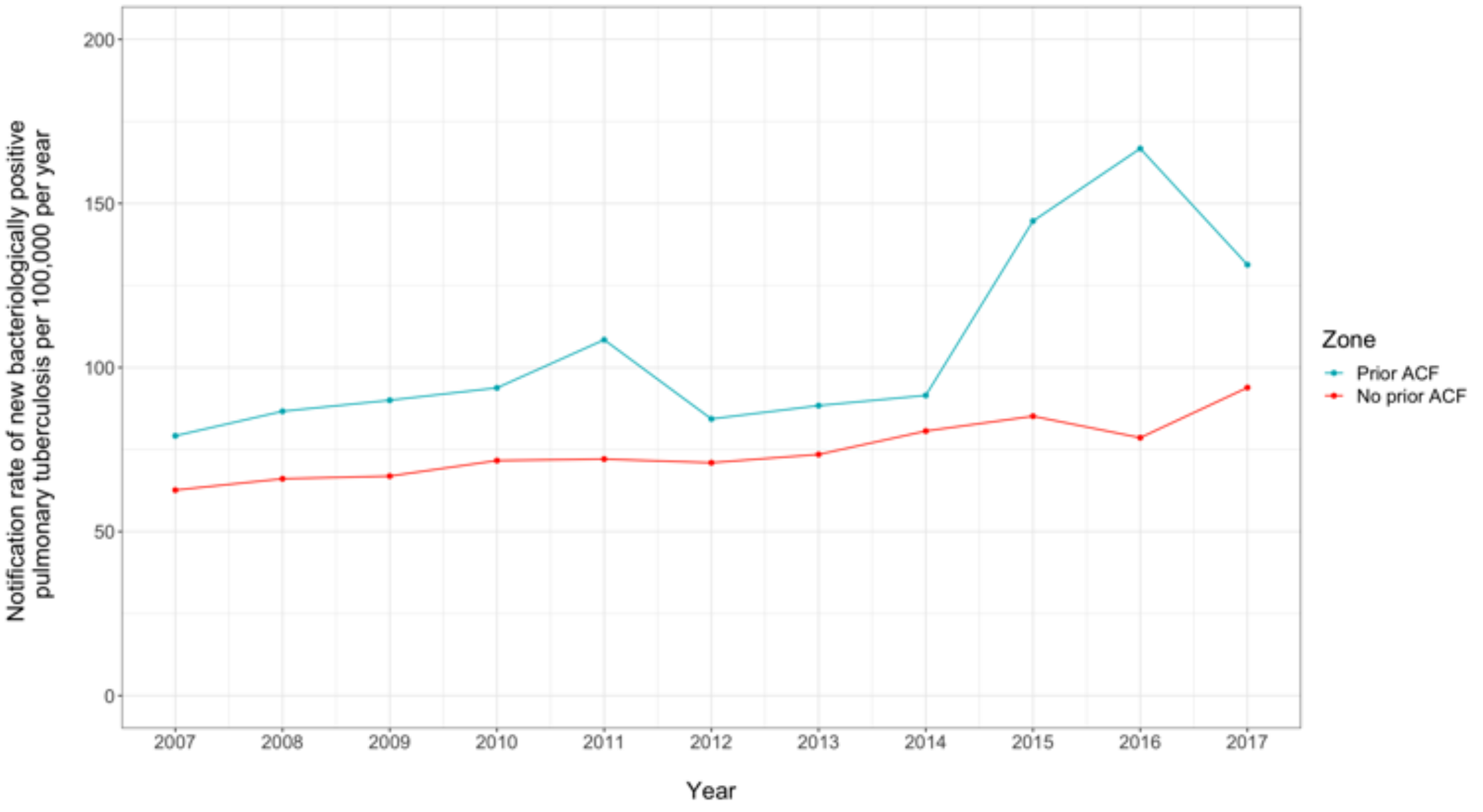

Supplement: S1 Fig — (TIFF) [file pgph.0002155.s003.tiff]

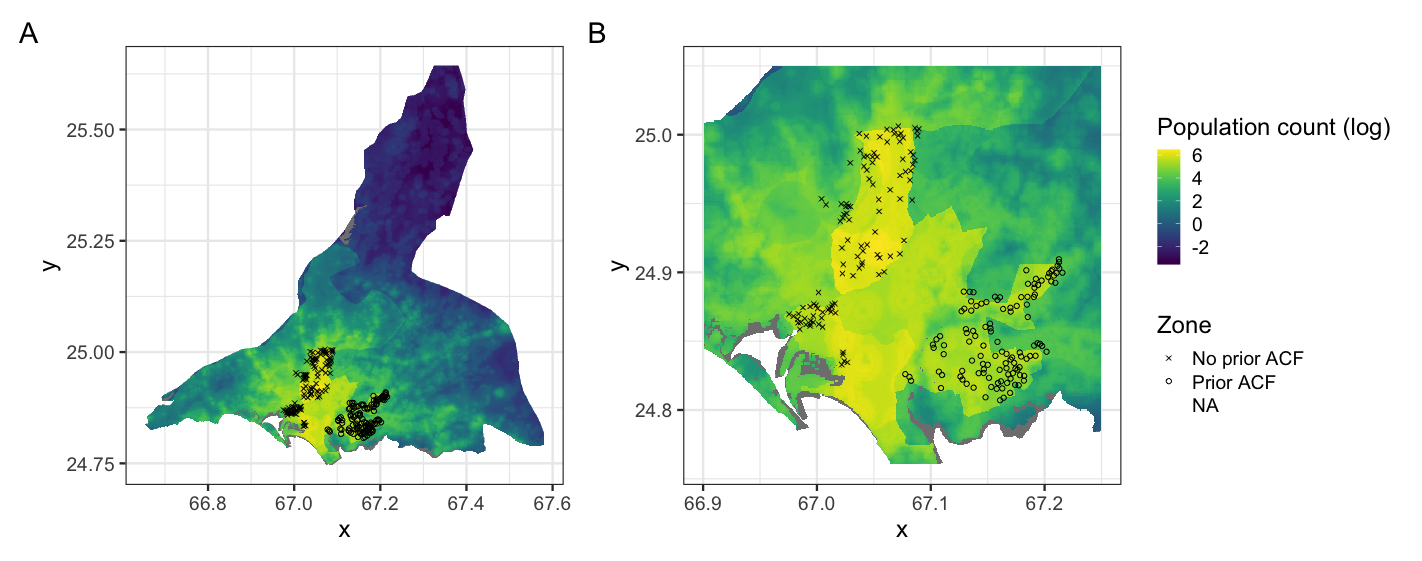

Supplement: S3 Fig — A = whole Karachi. B = map zoomed in to show distribution of sampling units in the two zones (no prior ACF [Karachi South, Central and West] and prior ACF [Karongi district]). The shapefile used to generate the map was obtained from Humanitarian Data Exchange (https://data.humdata.org/dataset/cod-ab-pak; administrative level 2 boundary shapefile “pak_admbnda_adm2_wfp_20220909.shp”), which is shared under Creative Common Attributions 4.0 International (CC by License). The population data for 2017 for Karachi displayed in the maps was obtained from WorldPop Hub (https://hub.worldpop.org/geodata/summary?id=28175) which is also shared under the Creative Commons Attribution 4.0 International License.[22]. (TIFF) [file pgph.0002155.s005.tiff]

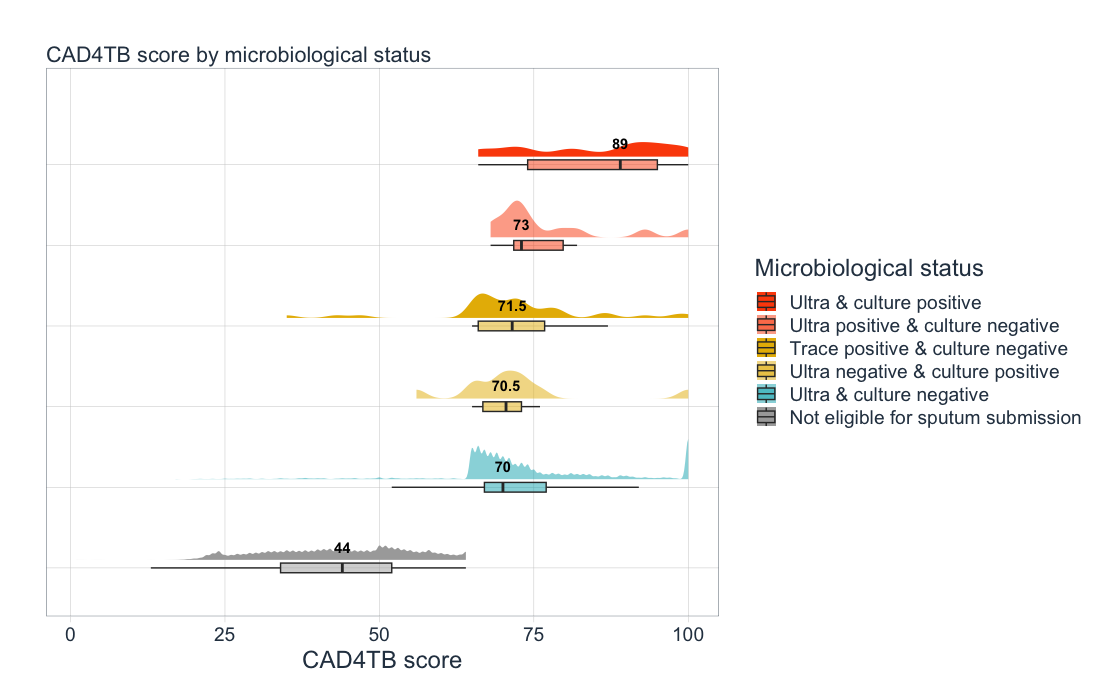

Supplement: S4 Fig — (TIFF) [file pgph.0002155.s006.tiff]
