# Supplementary material for: Insights into tuberculosis burden in Karachi, Pakistan: A concurrent adult tuberculosis prevalence and child Mycobacterium tuberculosis infection survey
Source: PLOS Glob Public Health. 2024 Aug 28;4(8):e0002155. doi: 10.1371/journal.pgph.0002155 (PMC11356439; doi:10.1371/journal.pgph.0002155)
Supplement: S1 Text — (DOCX) [file pgph.0002155.s007.docx]

**S1 Text. Models estimating adult tuberculosis prevalence**

*Model 1. Robust standard errors (complete case analysis)*

This model is restricted to survey participants with equal weight given to each individual in the sample. It excludes individuals for whom there was no microbiological result. This method therefore underestimates true prevalence amongst participants because data are missing for individuals who were eligible for sputum examination, who have a relative higher probability of being diagnosed with tuberculosis compared to those not eligible for sputum examination.

*Model 2. Robust standard errors and missing value imputation (for individuals eligible for sputum examination) with inverse probability weighting (applied to all survey participants)*

This model uses multivariate imputation by chained equations (MICE) for individuals who were eligible for sputum examination (e.g. CAD4TB score ≥65 or tuberculosis symptoms) but had missing Xpert Ultra and/or culture results. The MICE is performed on a variable-by-variable basis using a series of univariate imputation models, one for each incomplete variable. The univariate models are fitted iteratively with each variable imputed in turn, conditioning on the completely observed variables and the most recent imputed values of incomplete variables. This algorithm is then run multiple times in parallel to obtain 20 imputed datasets. Variables included in the imputation model were age, sex, cough, fever, night sweats, weight loss, CAD4TB score, Xpert Ultra and culture results, whether resident in a *katchi abaadi* and district. Inverse probability weighting was then applied to all survey participants to adjust for differentials in participation by age, sex and cluster with the aim of representing the whole of the survey eligible population. Summaries from eligibility through to microbiological results by district and sex are shown in supplementary table S1.

Models estimating child *M.tuberculosis* infection prevalence

*Model 1. Complete case analysis using non-parametric bootstrap sampling (with resampling)*

This model is based on non-parametric (resampling) bootstrap sampling of the complete case data for age and IGRA results to generate a median ARTI estimate with a 95% uncertainty interval using the percentile method.

*Model 2. Robust standard errors and missing value imputation of missing IGRA results with inverse probability weighting (applied to all survey participants)*

This model uses MICE (as described above) to impute missing IGRA results. Variables included in the imputation model were age, sex, cough, fever, night sweats, failure to thrive, known tuberculosis contact in last two years, neighbourhood block-level characteristics including number of adults, median CAD4TB score, number of people with pulmonary tuberculosis identified, whether resident in a *katchi abaadi*, district, *tehsil*, zone. Inverse probability weighting was then applied to all survey participants to adjust for differentials in participation by age, sex and cluster with the aim of representing the whole of the survey eligible population. The resulting point prevalence of *M.tuberculosis* infection and 95% confidence intervals were then used to estimate the ARTI and 95% confidence. Limitation here is that the uncertainty in the age distribution is not accounted for in the 95% confidence interval of the ARTI.
