# Supplementary material for: Insights into tuberculosis burden in Karachi, Pakistan: A concurrent adult tuberculosis prevalence and child Mycobacterium tuberculosis infection survey
Source: PLOS Glob Public Health. 2024 Aug 28;4(8):e0002155. doi: 10.1371/journal.pgph.0002155 (PMC11356439; doi:10.1371/journal.pgph.0002155)
Supplement: S1 Table — (DOCX) [file pgph.0002155.s008.docx]

**S1 Table. Xpert Ultra ‘trace positive’ only case series (n=38)**

| **District** | **Gender** | **Age** | **CAD4TB score** | **At least one TB symptom** | **Cough** | **Fever** | **Night sweats** | **Weight loss** | **Comment** |
| --- | --- | --- | --- | --- | --- | --- | --- | --- | --- |
| Korangi | F | 19 | 65 | 0 | 0 | 0 | 0 | 0 |  |
| Central | F | 45 | 66 | 0 | 0 | 0 | 0 | 0 |  |
| South | F | 50 | 66 | 0 | 0 | 0 | 0 | 0 |  |
| Korangi | F | 60 | 66 | 0 | 0 | 0 | 0 | 0 |  |
| Korangi | F | 52 | 66 | 0 | 0 | 0 | 0 | 0 |  |
| Central | M | 38 | 66 | 0 | 0 | 0 | 0 | 0 |  |
| Korangi | F | 50 | 66 | 0 | 0 | 0 | 0 | 0 | Culture-positive |
| South | F | 18 | 67 | 0 | 0 | 0 | 0 | 0 |  |
| Korangi | M | 40 | 68 | 0 | 0 | 0 | 0 | 0 |  |
| West | M | 15 | 68 | 0 | 0 | 0 | 0 | 0 |  |
| Central | F | 70 | 69 | 0 | 0 | 0 | 0 | 0 |  |
| Korangi | F | 50 | 69 | 0 | 0 | 0 | 0 | 0 |  |
| Korangi | F | 50 | 69 | 0 | 0 | 0 | 0 | 0 |  |
| Central | M | 72 | 71 | 0 | 0 | 0 | 0 | 0 |  |
| South | F | 50 | 72 | 0 | 0 | 0 | 0 | 0 |  |
| West | M | 60 | 72 | 0 | 0 | 0 | 0 | 0 |  |
| South | M | 35 | 72 | 0 | 0 | 0 | 0 | 0 |  |
| West | M | 17 | 73 | 0 | 0 | 0 | 0 | 0 |  |
| Korangi | F | 38 | 76 | 0 | 0 | 0 | 0 | 0 |  |
| Central | M | 60 | 77 | 0 | 0 | 0 | 0 | 0 |  |
| Korangi | F | 45 | 78 | 0 | 0 | 0 | 0 | 0 |  |
| Central | F | 70 | 79 | 0 | 0 | 0 | 0 | 0 |  |
| South | F | 50 | 79 | 0 | 0 | 0 | 0 | 0 |  |
| Central | M | 45 | 86 | 0 | 0 | 0 | 0 | 0 |  |
| Korangi | F | 65 | 87 | 0 | 0 | 0 | 0 | 0 | Previous TB more than 2 years ago |
| Korangi | F | 21 | . | 0 | 0 | 0 | 0 | 0 | Pregnant |
| Central | F | 21 | . | 0 | 0 | 0 | 0 | 0 | Pregnant |
| South | F | 22 | . | 0 | 0 | 0 | 0 | 0 | Pregnant |
| South | F | 16 | 35 | 1 | 1 | 0 | 0 | 1 |  |
| Central | M | 60 | 43 | 1 | 1 | 0 | 0 | 1 | Known TB contact last 2 years |
| Central | M | 35 | 47 | 1 | 0 | 1 | 1 | 0 |  |
| Korangi | F | 27 | 66 | 1 | 1 | 1 | 0 | 0 | Previous TB more than 2 years ago |
| South | M | 55 | 72 | 1 | 1 | 0 | 0 | 0 | Previous TB less than 2 years ago |
| Central | M | 55 | 73 | 1 | 1 | 0 | 0 | 1 |  |
| West | F | 55 | 74 | 1 | 1 | 0 | 0 | 0 |  |
| Central | F | 22 | 93 | 1 | 1 | 0 | 0 | 0 |  |
| Central | M | 40 | 98 | 1 | 0 | 0 | 0 | 1 |  |
| West | F | 25 | 100 | 1 | 1 | 0 | 0 | 0 | Previous TB less than 2 years ago |
