# Supplementary material for: Insights into tuberculosis burden in Karachi, Pakistan: A concurrent adult tuberculosis prevalence and child Mycobacterium tuberculosis infection survey
Source: PLOS Glob Public Health. 2024 Aug 28;4(8):e0002155. doi: 10.1371/journal.pgph.0002155 (PMC11356439; doi:10.1371/journal.pgph.0002155)
Supplement: S2 Table — (DOCX) [file pgph.0002155.s009.docx]

**S2 Table. Characteristics of participants and microbiological outcomes of the adult pulmonary tuberculosis survey stratified by district and sex**

|  | **Karachi Central** | | | **Karachi South** | | | **Karachi West** | | | **Korangi** | | |
| --- | --- | --- | --- | --- | --- | --- | --- | --- | --- | --- | --- | --- |
|  | **Male** | **Female** | **Overall** | **Male** | **Female** | **Overall** | **Male** | **Female** | **Overall** | **Male** | **Female** | **Overall** |
| Eligible (N = 44,565) | 4,305 | 4,190 | 8,495 | 3,185 | 3,202 | 6,387 | 3,701 | 3,714 | 7,415 | 11,301 | 10,967 | 22,268 |
| Consented to participate  (N = 34,964) | 2,843 | 3,720 | 6,563 | 2,029 | 2,885 | 4,914 | 2,122 | 3,327 | 5,449 | 8,072 | 9,966 | 18,038 |
| % participation | 66·0% | 88·8% | 77·3% | 63·7% | 90·1% | 76·9% | 57·3% | 89·6% | 73·5% | 71·4% | 90·9% | 81·0% |
| Age (years),  mean (sd) | 34·0 (15·5) | 32·9 (14·2) | 33·4 (14·8) | 34·2 (15·9) | 34·5 (14·8) | 34·4 (15·2) | 32·6 (15·2) | 32·5 (14·4) | 32·5 (14·7) | 33·5 (15·2) | 33·3 (14·3) | 33·4 (14·7) |
| Symptom status |  |  |  |  |  |  |  |  |  |  |  |  |
| Any cough (n=34,896) | 316 (10·4%) | 191 (4·8%) | 507 (7·2%) | 182 (9·0%) | 183 (6·4%) | 365 (7·4%) | 102 (5·3%) | 126 (4·2%) | 228 (4·6%) | 689 (8·6%) | 661 (6·6%) | 1350 (7·5%) |
| Cough ≥ 2 weeks (n=34,896) | 35 (1·2%) | 27 (0·7%) | 62 (0·9%) | 35 (1·7%) | 40 (1·4%) | 75 (1·5%) | 5 (0·3%) | 9 (0·3%) | 14 (0·3%) | 61 (0·8%) | 77 (0·8%) | 139 (0·8%) |
| Any TB symptom^ | 51 (1·7%) | 42 (1·1%) | 93 (1·3%) | 45 (2·2%) | 70 (2·4%) | 115 (2·3%) | 8 (0·4%) | 16 (0·5%) | 24 (0·5%) | 100 (1·2%) | 133 (1·3%) | 233 (1·3%) |
| Sought medical care if symptoms (n=451) | 13 (25·5%) | 22 (52·4%) | 35 (37·6%) | 4 (9·1%) | 16 (28·1%) | 20 (19·8%) | 0 (0·0%) | 3 (18·8%) | 3 (12·5%) | 30 (30·0%) | 47 (35·3%) | 77 (33·1%) |
| On TB treatment (n=34,875) | 13 (0·4%) | 4 (0·1%) | 17 (0·2%) | 1 (0·1%) | 5 (0·2%) | 6 (0·1%) | 2 (0·1%) | 5 (0·2%) | 7 (0·1%) | 23 (0·3%) | 22 (0·2%) | 45 (0·3%) |
| Previous history of TB (n=34,831) | 49 (1·6%) | 76 (1·9%) | 125 (1·8%) | 41 (2·0%) | 91 (3·2%) | 132 (2·7%) | 23 (1·2%) | 68 (2·3%) | 91 (1·8%) | 94 (1·2%) | 197 (2.0%) | 291 (1·6%) |
| Known TB contact within last 2 years (n=34,807) | 80 (2·6%) | 144 (3·6%) | 224 (3·2%) | 63 (3·1%) | 106 (3·7%) | 169 (3·5%) | 37 (1·9%) | 114 (3·8%) | 151 (3·1%) | 175 (2·2%) | 308 (3·1%) | 483 (2·7%) |
| Self-reported having diabetes (n=34,332) | 87 (2·9%) | 190 (4·8%) | 277 (4·0%) | 44 (2·2%) | 113 (4·0%) | 157 (3·2%) | 31 (1·6%) | 94 (3·1%) | 125 (2·6%) | 231 (2·9%) | 421 (4·3%) | 652 (3·7%) |
| Smoking status |  |  |  |  |  |  |  |  |  |  |  |  |
| Ex-smoker | 22 (0·7%) | 3 (0·1%) | 25 (0·0%) | 18 (0·9%) | 2 (0·1%) | 20 (0·0%) | 17 (0·9%) | 4 (0·1%) | 21 (0·4%) | 52 (0·6%) | 10 (0·1%) | 62 (0·3%) |
| Currently smoking | 397 (13·1%) | 18 (0·5%) | 415 (5·9%) | 216 (10·7%) | 98 (3·4%) | 314 (6·4%) | 168 (8·7%) | 61 (2·0%) | 229 (4·6%) | 1057 (13·1%) | 44 (0·4%) | 1101 (6·1%) |
| CXR in last 3 months (n=31,175) | 25 (0·9%) | 36 (1·1%) | 61 (1·0%) | 17 (0·9%) | 15 (0·6%) | 32 (0·7%) | 17 (1·0%) | 12 (0·5%) | 29 (0·6%) | 80 (1·1%) | 87 (1·0%) | 167 (1·0%) |
| Self-reported pregnancy (n=19,801) | - | 104 (2·6%) | - | - | 66 (2·3%) | - | - | 110 (3·6%) | - | - | 281 (2·8%) | - |
| Self-reported disability | 6 (0·2%) | 14 (0·4%) | 20 (0·3%) | 6 (0·3%) | 10 (0·4%) | 16 (0·3%) | 3 (0·2%) | 3 (0·1%) | 6 (0·1%) | 17 (0·2%) | 24 (0·2%) | 41 (0·2%) |
| Digital chest x-ray (n=30,892) | | | | | | | | | | | | |
| Number x-rayed (% of consented) | 2820 (92·9%) | 3328 (83·2%) | 6158 (87·4%) | 1910 (94·1%) | 2445 (84·8%) | 4355 (88·6%) | 1761 (91·3%) | 2477 (81·6%) | 4328 (85·4%) | 7573 (93·8%) | 8568 (86·0%) | 16141 (89·5%) |
| Median CAD4TB score (IQR) | 46 (35 – 56) | 47 (38 – 55) | 47 (36 – 55) | 46 (34 – 57) | 48 (37 – 55) | 47 (36 –56 ) | 47 (36 – 56) | 46 (36 – 55) | 46 (36 – 55) | 44 (33 – 55) | 46 (36 – 54) | 45 (35 – 55) |
| CAD4TB ≥ 65 (% x-rayed) | 312 (11·1%) | 264 (7·9%) | 576 (9·4%) | 219 (11·5%) | 210 (8·6%) | 429 (9·9%) | 210 (11·9%) | 204 (8·2%) | 414 (9·8%) | 729 (9·6%) | 658 (7·7%) | 1387 (8·6%) |
| **Sputum submission eligibility (n=34,946)** | | | | | | | | | | | | |
| Unable to establish eligibility due to missed CXR  (n = 3,111) | 178 (6·3%) | 485 (13·0%) | 663 (10·1%) | 97 (4·8%) | 336 (11·7%) | 433 (8·8%) | 154 (7·3%) | 465 (14·0%) | 619 (11·3%) | 397 (4·9%) | 999 (10·0%) | 1396 (7·7%) |
| Not eligible for sputum submission  (n = 28,018) | 2,330 (82·0%) | 2,846 (76·5%) | 5,176 (78·9%) | 1,675 (82·6%) | 2,185 (75·7%) | 3,860 (78·6%) | 1,733 (81·6%) | 2,513 (75·5%) | 4,246 (77·9%) | 6,849 (84·9%) | 7,887 (79·2%) | 14,736 (81·7%) |
| Eligible for sputum submission* (n=3,835 ) | 335 (11·7%) | 389 (10·5%) | 724 (11·0%) | 257 (12·7%) | 364 (12·6%) | 621 (12·6%) | 235 (11·1%) | 349 (10·5%) | 584 (10·7%) | 826 (10·2%) | 1080 (10·8%) | 1906 (10·6%) |
| Unable to expectorate  (n=439) | 42 (12·5%) | 41 (10·5%) | 83 (11·5%) | 35 (13·6%) | 69 (19·0%) | 104 (16·8%) | 20 (8·5%) | 26 (7·5%) | 46 (7·9%) | 100 (12·1%) | 106 (9·8%) | 206 (10·8%) |
| Submitted at least one sample (n=2,599) | 217 (64·8%) | 252 (64·8%) | 469 (64·8%) | 156 (60·7%) | 213 (58·5%) | 369 (59·4%) | 153 (65·1%) | 234 (67·1%) | 387 (66·3%) | 584 (71·2%) | 790 (74·4%) | 1374 (73·0%) |
| *All samples rejected by lab* (n=34) | 3 (0·9%) | 4 (1·0%) | 7 (1·0%) | 7 (2·7%) | 3 (0·8%) | 10 (1·6%) | 1 (0·4%) | 1 (0·3%) | 2 (0·3%) | *10 (1·2%)* | *5 (0·5%)* | *15 (0·8%)* |
| **Microbiological status if at least one sputum sample tested (n=2,565)** | | | | | | | | | | | | |
| Valid result for Xpert Ultra (% of eligible for sputum submission) | 212 (63·3%) | 241 (62·0%) | 453 (62·6%) | 144 (56·0%) | 208 (57·1%) | 352 (56·7%) | 151 (64·3%) | 232 (66·5%) | 383 (65·6%) | 562 (68·0%) | 766 (70·9%) | 1328 (69·7%) |
| Valid result for culture  (% of eligible for sputum submission) | 217 (64·8%) | 251 (64·5%) | 468 (64·6%) | 156 (60·7%) | 213 (58·5%) | 369 (59·4%) | 153 (65·1%) | 234 (67·0%) | 387 (66·3%) | 584 (70·7%) | 790 (73·1%) | 1374 (72·0%) |
| Xpert Ultra & culture negative | 199 | 236 | 435 | 140 | 203 | 343 | 146 | 229 | 375 | 557 | 762 | 1319 |
| Any form of microbiological confirmation  (% of at least one sputum submitted) | 15 (6·9%) | 12 (4·8%) | 27 (5·8%) | 9 (5·8%) | 7 (3·3%) | 16 (4·3%) | 6 (3·9%) | 4 (1·7%) | 10 (2·6%) | 17 (2·9%) | 23 (2·9%) | 40 (2·9%) |
| Xpert Ultra-negative & culture positive | 1/15 (6·7%) | 2/12 (16·7%) | 3/27 (11·1%) | 1/9 (11·1%) | 0/7 (0·0%) | 1/16 (6·3%) | 0/6 (0·0%) | 0/4 (0·0%) | 0/10 (0·0%) | 2/17 (11·8%) | 6/23 (26·0%) | 8/40 (20·0%) |
| Xpert Ultra ‘trace positive’ only ** | 8/15 (53·3%) | 5/12 (41.6%) | 13/27 (48·1%) | 2/9 (22·2%) | 6/7 (85·7%) | 8/16 (50·0%) | 3/6 (50·0%) | 2/4 (50·0%) | 5/10 (50·0%) | 1/17 (5·9%) | 10/23 (43·5%) | 11/40 (27·5%) |
| Xpert Ultra-positive & culture negative | 4/15 (26·7%) | 0/12 (0·0%) | 4/27(14·8%) | 0/16 (0·0%) | 0/16 (0·0%) | 0/16 (0·0%) | 1/6 (16·7%) | 0/4 (0·0%) | 1/10 (10·0%) | 4/17 (23·5%) | 4/23 (17·4%) | 8/40 (20·0%) |
| Xpert Ultra-positive & culture positive | 2/15 (13·3%) | 5/12 (41·6%) | 7/27 (25·9%) | 6/9 (66·7%) | 1/7 (14·3%) | 7/16 (43·7%) | 2/6 (33·3%) | 2/4 (50·0%) | 4/10 (40·0%) | 10/17 (58·8%) | 3/23 (13·0%)*** | 13/40 (32·5%) |
| **Follow-up and treatment status if microbiologically positive sputum sample (n=93)** | | | | | | | | | | | | |
| Unable to contact | 2 | 3 | 5 (18·5%) | 3 | 2 | 5 (31·3%) | 1 | 1 | 2 (20·0%) | 7 | 4 | 11 (27·5%) |
| Contacted and refused follow-up | 5 | 6 | 11 (40·8%) | 2 | 4 | 6 (37·5%) | 3 | 2 | 5 (50·0%) | 3 | 5 | 8 (20·0%) |
| Reviewed clinically and TB excluded | 4 | 1 | 5 (18·5%) | 0 | 1 | 1 (6·2%) | 1 | 0 | 1 (10·0%) | 0 | 9 | 9 (22·5%) |
| Started treatment | 4 | 2 | 6 (22·2%) | 4 | 0 | 4 (25·0%) | 1 | 1 | 2 (20·0%) | 7 | 5 | 12 (30·0%) |

* Eligibility for sputum included all those who screened positive on symptoms or CAD4TB≥65 or were disabled or pregnant (no chest x-ray) or on TB treatment

** culture-negative

*** includes ‘trace positive’, culture-positive (n=1)
