# Supplementary material for: Insights into tuberculosis burden in Karachi, Pakistan: A concurrent adult tuberculosis prevalence and child Mycobacterium tuberculosis infection survey
Source: PLOS Glob Public Health. 2024 Aug 28;4(8):e0002155. doi: 10.1371/journal.pgph.0002155 (PMC11356439; doi:10.1371/journal.pgph.0002155)
Supplement: S3 Table — (DOCX) [file pgph.0002155.s010.docx]

**S3 Table. Sensitivity analysis excluding Xpert Ultra ‘trace positive’ culture negative cases**

| **Excluding all Xpert Ultra ‘trace positive’ culture negative***  **N=56** | Overall point prevalence  (95% CI) | **No prior ACF** | | | **Prior ACF** | | |
| --- | --- | --- | --- | --- | --- | --- | --- |
|  |  | Male | Female | Overall | Male | Female | Overall |
| Cluster-level |  |  | | |  | | |
| N = 13**  (n=9 ; n=4) | 167  (115 – 242) | 272  (156 – 477) | 116  (67 – 204) | 179  (113 – 284) | 172  (71 – 422) | 112  (46 – 275) | 144  (72 – 287) |
| Including sampling weights | 165  (114 – 239) | 257  (146 – 456) | 118  (64 – 216) | 168  (111 – 254) | 172  (63 – 472) | 112  (31 – 413) | 143  (73 – 280) |
| Individual-level |  |  | | |  | | |
| Complete case  N = 30,546  (n=14,462 ; n= 16,084) | 183  (131 – 236) | 272  (140 – 404) | 121  (0 – 246) | 187  (125 – 247) | 216  (131 – 300) | 150  (27 – 272) | 180  (97 – 263) |
| IPW/MI  N = 31,816  (n=15,185 ; n=16,631) | 231  (163 – 298) | 311  (147 – 474) | 147  (20 – 272) | 232  (146 – 318) | 274  (150 – 397) | 171  (45 – 296) | 225  (129 – 321) |

IPW/MI inverse probability weighting and multiple imputation using chained equations

ACF active case finding CI confidence interval

*Excludes 11 cases (1 male and 10 female) from the prior ACF zone and 26 cases (13 male and 13 female) from the no prior ACF

**Clusters relate to the 13 tehsils (9 in the no prior ACF zone and 4 in the prior ACF zone)
