# Supplementary material for: Insights into tuberculosis burden in Karachi, Pakistan: A concurrent adult tuberculosis prevalence and child Mycobacterium tuberculosis infection survey
Source: PLOS Glob Public Health. 2024 Aug 28;4(8):e0002155. doi: 10.1371/journal.pgph.0002155 (PMC11356439; doi:10.1371/journal.pgph.0002155)
Supplement: S4 Table — (DOCX) [file pgph.0002155.s011.docx]

**S4 Table. Adult pulmonary tuberculosis prevalence estimates stratified by zone and *katchi abadi* status (per 100,000 population)**

|  | No prior ACF | | Prior ACF | |
| --- | --- | --- | --- | --- |
|  | n/N* | Point prevalence estimates using IPW/MI model  (95% CI) | n/N* | Point prevalence estimates using IPW/MI model  (95% CI) |
| *Katchi abadi* | 8/1,287 | 640  (132 – 1147) | 1/1,199 | 77  (0 – 250) |
| Non *katchi abadi* | 43/13,199 | 313  (202 – 424) | 37/14,894 | 246  (120 – 372) |

IPW inverse probability weight MI multiple imputation CI confidence interval ACF active case finding

*Complete case numbers
