# Supplementary material for: Insights into tuberculosis burden in Karachi, Pakistan: A concurrent adult tuberculosis prevalence and child Mycobacterium tuberculosis infection survey
Source: PLOS Glob Public Health. 2024 Aug 28;4(8):e0002155. doi: 10.1371/journal.pgph.0002155 (PMC11356439; doi:10.1371/journal.pgph.0002155)
Supplement: Attachment — Submitted filename: Response to Reviewers.docx [file pgph.0002155.s012.docx]

Response to Reviewers

Re: PGPH-D-23-00926

Dear Editorial team,

We appreciate the time the editorial team and the reviewers have taken to peer review our manuscript entitled *"Insights into tuberculosis burden in Karachi, Pakistan: a concurrent adult tuberculosis prevalence and child M. tuberculosis infection survey"* which we think has strengthened the manuscript*.*

Based on the comments by both Reviewer #1 and #2 we have dropped Ultra trace positive culture-negative with a previous history of tuberculosis (n=4) from the case definition for the main analysis and have included a sensitivity analysis excluding all Ultra trace positive culture negative cases.

We have addressed the comments (in blue italics below) and provide a point-by-point response highlighting where changes have occurred in the revised manuscript.

Reviewer #1:

Many thanks for taking the time to review our manuscript.

*1. In the title “M.tuberculosis” should be “Mycobacterium tuberculosis”. In the manuscript (MS) the authors abbreviate “Mycobacterium tuberculosis” as “M. tuberculosis” without a space, but thereafter inconsistently add or delete the space – the correct abbreviation is “M. tuberculosis” (with space between M. and tuberculosis)*

We have changed the title to include *Mycobacterium tuberculosis* and used *M. tuberculosis* throughout the manuscript.

*2. Abstract: Suggest add: “Pakistan” after “Karachi”*

We have added Pakistan after Karachi.

*3. Abstract and MS: Spelling of “bacteriologically-confirmed” pulmonary tuberculosis – inconsistent in abstract and MS with/without hyphen, but correct spelling is without a hyphen in this wording even though it is followed by a noun*

We have removed the hyphen through the abstract and manuscript between bacteriologically confirmed.

*4. Methods, Study design and setting, last line: Should use “ACF” as already previously abbreviated in MS*

We have changed to ACF in the last line of Methods, Study design and setting

*5. Methods, Participants, line 9: Suggest state that adults are for this study defined as ≥ 15 years of age: “All adults, defined for this study as aged ≥15 years who were resident…”. The question is whether in Pakistan the 15-<18-year-old participants can give their own consent for study participation or whether their parents/legal guardians should give consent, even though verbal as in this study?*

For this study, individuals aged 15-17 years were able to give their own consent for study participation.

*6. Study procedures, line 14: Suggest add “cultures” after “liquid and solid media”*

We have added “cultures”.

*7. Child M. tuberculosis infection survey using interferon-gamma release assays (IGRA), line 9: suggest “a 6-month course…”*

We have made the suggested change to “6-month course”.

*8. Page 6, under “Statistical methods” there is a subheading “Statistical analysis” followed by a paragraph with subheading “Child survey case definition”. The reviewer thinks that, because the paragraph following “Child survey case definition” is again about the adults, it would be much better that the “Child survey case definition” moves one paragraph further down t keep adults and children’ data analyses together.*

We agree that it would be better than the “Child survey case definition” moves one paragraph further down and have made that change.

*9. Page 6, second to last paragraph, last line: The missing IGRA data in children “(n=1,119)” falls somewhere out of the blue – should this not be mentioned in the results?*

We agree that “(n=1,119)” in the Statistical methods section does not make sense and have removed it from here. It is clear from Figure S2b study flowchart that the missing data on IGRA result is n=1,119.

*10. Results, Adults: I understand that the authors had a specific definition for “microbiologically confirmed pulmonary tuberculosis”, but does CAD4TB clearly distinguish between old scarring of TB and “active” PTB, and could other conditions be similar to TB on CAD4TB?*

In our survey, CAD4TB was used to triage for sputum submission rather than as a TB diagnostic test. However, this question is pertinent as computer-aided detection software, such as CAD4TB, has been found to have poor ability to differentiate between old scarring and active lesions.^1^

A recent study by Kagujje *et al.*^2^ assessing the performance of CAD software for triage of active tuberculosis among persons with a history of previous tuberculosis found that the optimal CAD threshold for CAD4TB detecting active tuberculosis differed substantially by previous tuberculosis history: 45 in a HIV-negative population with a history of previous TB and 18 in a HIV-negative population with no history of previous TB. Our cut-off of CAD4TB≥65 is substantially higher than 45 and should account for lower specificity of CAD software in those with a previous history of tuberculosis. In our study population, the median and IQR of CAD4TB for those with a previous history of TB (excluding those with some form of microbiological confirmation) was 54 (IQR: 42 – 65) and in those with no previous history of TB was 45 (IQR: 35 – 54).

The review of literature on different CAD thresholds has highlighted that the high cut-off of CAD4TB (CAD4TB≥65) used in our survey may actually have resulted in an overall underestimation of microbiologically confirmed pulmonary tuberculosis. We would appreciate Reviewer #1 thoughts on this.

Of note, the proportion of screened individuals with a previous tuberculosis history was only 1.8% in our survey although was higher in the non-ACF zone (2.1%) than in the ACF zone (1.6%).

*It seems that most adults diagnosed with PTB either did not have PTB or did not consider themselves as having PTB needing treatment – could this be an overestimation due to “M. tuberculosis infection” or previous PTB rather than “active” TB disease?*

Our experience as the survey team was that the majority of adults diagnosed with PTB did not consider themselves as having PTB needing treatment because they did not exhibit any of the symptoms or feel unwell. Indeed some of the clinicians at the local hospital reviewing the patients also did not recognise ‘asymptomatic’ PTB as requiring treatment.

As noted by Esmail et al. the increased use of CXR screening as part of ACF interventions has resulted in TB programmes identifying “greater numbers of individuals that are not comfortably managed within existing treatment algorithms” having been diagnosed with subclinical disease.^3^ Our interpretation of these data is that we identified subclinical disease rather than *M. tuberculosis* infection as the majority of the Ultra trace only cases had a CAD4TB≥65 and no history of previous tuberculosis.

*This is somewhat difficult to interpret in this study – how do these “cases” that seem not to have “disease” effect the two zones (prior and non-prior ACF), if left out?*

We agree that it is important to show the effect of excluding these ‘cases’ on prevalence estimates of the prior and non-prior ACF zones and have therefore included a sensitivity analysis. We have also added a case series table to the supplementary data describing all the Xpert Ultra trace cases.

*11. Results: A concern regarding the prevalence of infection amongst the children is the very low inclusion/interpretable IGRA result “rate” – only 1392/2511 (55.4%) – do the authors think that this could be an important bias in the study? What were the main reasons for refusal of participation? Interesting that contact with TB in the past two years was higher in the prior ACF group and IGRA-positivity lower – comment?*

We do think that the participation rate of 55% is low and is likely to have resulted in bias, especially if risk of IGRA-positivity is systematically different in those that participated versus those that did not. We have added a sentence to the discussion on page 11, 2^nd^ paragraph. Of note, 20% of children did not participate due to unavailability of the male guardian to give consent which was the same in the both the ACF and non-ACF group. We did not collect reason for refusal to participate.

We also found it of interest that reported contact with TB in the past two years was higher in the prior ACF group and IGRA-positivity lower. This would only be a speculative comment and hence was not included in the discussion but we considered whether this could be due to the ongoing ACF programmes in Korangi resulting in more TB identified but diagnosed earlier so overall reduced duration of infectiousness so less transmission. If reviewer 1 thinks that such a speculative comment should be included in the discussion, we would be happy to comply but have refrained for now.

*12. Discussion, page 10, 4th paragraph, line 3: “Of note, only 20% of those who were…” – this is not the same as in the results section which indicates 26% - which is correct?*

The 26% is the correct and we have changed the sentence in the Discussion, page 10, 4^th^ paragraph.

*13. Finally, there is no mention of HIV positivity – is this an issue at all in Karachi?*

Prevalence of HIV among pulmonary tuberculosis patients is 0.29% in Sindh.^4^ We do not think that one can say HIV is not an issue in Karachi as prevalence is increasing in specific high risk groups (e.g. male sex workers and transgender women) however HIV-associated tuberculosis only accounts for <1% of the TB burden in Pakistan.

Reviewer #2:  *While it is an important study, there are two major issues that need to be addressed before it can be accepted for publication.*

Many thanks for taking the time to review our manuscript. Addressing these comments have definitely improved the manuscript and also highlighted key areas for further research in this field.

*Major issues
1. The inclusion of Xpert MTB/RIF Ultra trace only as cases in the adult prevalence survey case definition is problematic.

A total of 93 probable and definite bacteriologically confirmed pulmonary tuberculosis cases were included in the analysis. Of these 93 ‘cases’, 40% (37/93) were included on the basis of only an Xpert Ultra trace result. While no participants with Xpert Ultra trace were culture positive, the number that were culture negative is not reported.*

In table 1, the footnote ** states that none of the Xpert Ultra trace positive only group were culture positive. The single Xpert Ultra trace culture-positive microbiologically confirmed case is included in the Xpert Ultra positive & culture positive group. To be explicit, there were n=38 cases which were Xpert Ultra trace; n=37 were culture-negative and n=1 was culture-positive.

The decreased specificity of the Xpert Ultra test compared to the Xpert MTB/RIF has been well described, particularly if trace results are regarded as positive, and in patients with a recent prior history of TB.

Of the 93 cases identified, n=9 had a previous history of TB: of these, n=4 were Xpert Ultra trace positive only, n=4 were Xpert positive only (very low or low) and n=1 was Xpert Ultra positive and culture positive. Of note the majority of Xpert Ultra trace cases had a high CAD4TB score (≥65) and no previous history of tuberculosis. We excluded the 4 Xpert Ultra trace only with history of previous tuberculosis from the prevalence estimate analyses.

The proportion of cases that were Xpert Ultra trace only was substantially higher in the non-ACF ‘cases’ (26/53, 49%) compared to the ACF (11/40, 27.5%). Excluding these Ultra trace only results from the case definition would almost entirely remove the observed difference in the adult TB prevalence between the ACF and non-ACF areas. The Ultra trace only ‘cases’ therefore drive the reported difference in the adult pulmonary TB prevalence estimates, one of the central findings of the study.

We agree that the Ultra trace only ‘cases’ drive the reported differences and we have explicitly stated that in the discussion. However, we have used a high cut-off of CAD4TB for eligibility for sputum submission (CAD4TB>65) and therefore the majority of these Ultra trace only cases have a relatively abnormal CXR suspicious for TB.

To aid interpretation of the Ultra trace only group, we have included case series table to the supplementary material. Of note, 3/38 Ultra trace cases were in young pregnant women; 3/38 were symptomatic with a CAD4TB<65, one of whom had known contact with TB in last 2 years; 3/38 were symptomatic with a CAD4TB score of >90, only one of whom had a previous history of TB and 12/38 had a CAD4TB>70 of whom only one had a previous history of tuberculosis more than 2 years ago.

Furthermore, amongst the 40 ‘cases’ that were clinically assessed, TB was excluded in 16 participants with 12 of these 16 having trace only results. Once again calling into question the appropriateness of including Ultra trace only in the case definition.

This study was conducted at a time when there was no guidance on how to manage Ultra trace only results. Clinical assessment did not include repeat sputum testing. As noted by Reviewer 1 what might have been detected is something on the spectrum from *M. tuberculosis* infection to active clinical disease. Our interpretation is that we have detected a significant burden of subclinical disease.

This does however raise the question of whether these cases of subclinical disease should be included in prevalence estimates. We have added a sentence to the discussion highlighting this point. Our understanding is that the WHO Lime Handbook on conducting TB prevalence surveys is due to be updated very soon to help address some of these issues that have arisen since the roll-out of computer-aided detection of radiological abnormalities compatible with tuberculosis and more sensitive diagnostics in the last decade.

Lastly, the inclusion of the Ultra trace only participants in the case definition is not in keeping with the primary or secondary analysis outlined in the published protocol. The protocol includes the following case definitions for the adult pulmonary TB prevalence survey component:

a. “Definite bacteriologically confirmed pulmonary tuberculosis case is defined as study participant with one culture positive sputum specimen and at least one of the following conditions: Xpert Ultra-positive [or] Culture-positive (either LJ or MGIT) in another specimen.”
b. “Probable bacteriologically confirmed pulmonary tuberculosis case is defined as a study participant with an Xpert Ultra-positive sputum specimen but culture-negative on one or both sputum samples or a study participant with a single culture- positive sputum where laboratory cross-contamination has been excluded.” The protocol notes that “a sensitivity analysis using the ‘probable TB case’ definition will also be undertaken.” However, neither the definite nor the probable definitions include Ultra trace only in the definition.
Recommendation: The authors revise the case definition to be in keeping with the probable case definition in the protocol and excluding the trace only ‘cases’.

At the time of writing the published protocol, we considered the definition of Xpert Ultra-positive to include Xpert Ultra trace. This is supported by WHO guidance published in 2020 recommending that Xpert Ultra ‘trace call’ be considered a true positive in HIV-negative persons without a prior TB episode or recent history of TB treatment.^5^ ^6^ We have now included a sensitivity analysis which drops all Ultra trace culture-negative cases. The difference in prevalence estimates between the ACF zones is no longer apparent and a paragraph to the discussion has been added to discuss these findings.

2. Selection of the three non-ACF districts: While the rationale for the selection of the prior ACF district is well described in the manuscript, it appears that the 3 no prior ACF districts were chosen on the basis of convenience. Substantial spatial heterogeneity in TB burden has been well described in many cities, and thus is an alternative plausible explanation for the differences.

We agree that spatial heterogeneity in TB burden is often observed and might be an alternative plausible explanation for the differences observed. Interestingly, the proportion of study participants screened by digital CXR who had a CAD4TB score ≥ 65 was higher in all three non-ACF districts than in Korangi district (ACF zone) as shown in Table S1 in the supplementary material.

There is no striking heterogeneity observed in the clinical characteristics, sputum submission eligibility and microbiological status of study participants from the three non-ACF districts but we agree that it is important to show these data.

The higher average population within 100m in the non-prior ACF zone could potentially account for some of the difference in the paediatric IGRA prevalence and annual risk of infection. While the number of adults in neighbourhood blocks was included in the multiple imputation of missing IGRA results, indicating that the authors identified this as an important factor, it does not appear that the estimates were adjusted for district level population differences.

Recommendation: Authors should consider including district (or preferably neighbourhood block) characteristics in the analysis of the ARTI, and/or include discussion of alternative explanations.

In order to assess the effect of adjusting for average population count within 100 metres of each neighbourhood block we included the variable to the multiple imputation model. We found that adjusting for average population count within 100 metres does not qualitatively change the ARTI although it does widen the confidence intervals (ARTI 1.1% [95% CI: 0.1 – 2.1] for the no prior ACF zone and 0.7% [95% CI: 0.09 – 1.3] in the prior ACF zone) and have therefore not been included in the results. If reviewer #2 feels that this is an important finding then we will add it to the supplementary material.

Minor issue
*3. Providing a supplementary table similar to table 1 with the data disaggregated by the 4 districts (as opposed to providing aggregated data for the 3 non ACF districts) would assist the reader to assess whether there was substantial heterogeneity across the 3 non ACF districts.*

An additional supplementary table (Table S1) has been added with data disaggregated by the 4 districts.

**References**

1.	Qin, Z. Z. *et al.* Tuberculosis detection from chest x-rays for triaging in a high tuberculosis-burden setting: an evaluation of five artificial intelligence algorithms. *Lancet Digit. Health* **3**, e543–e554 (2021).

2.	Kagujje, M. *et al.* The Performance of Computer-Aided Detection Digital Chest X-ray Reading Technologies for Triage of Active Tuberculosis Among Persons With a History of Previous Tuberculosis. *Clin. Infect. Dis.* **76**, e894–e901 (2023).

3.	Esmail, H., Macpherson, L., Coussens, A. K. & Houben, R. M. G. J. Mind the gap – Managing tuberculosis across the disease spectrum. *EBioMedicine* **78**, 103928 (2022).

4.	Hasnain, J. *et al.* Screening for HIV among tuberculosis patients: a cross-sectional study in Sindh, Pakistan. *BMJ Open* **2**, e001677 (2012).

5.	World Health Organization. WHO consolidated guidelines on tuberculosis: Module 3: diagnosis – rapid diagnostics for tuberculosis detection. (2020).

6.	Chilukutu, L. *et al.* Prevalence and interpretation of Xpert® Ultra trace results among presumptive TB patients. *Public Health Action* **12**, 28–33 (2022).
