# Supplementary material for: Insights into tuberculosis burden in Karachi, Pakistan: A concurrent adult tuberculosis prevalence and child Mycobacterium tuberculosis infection survey
Source: PLOS Glob Public Health. 2024 Aug 28;4(8):e0002155. doi: 10.1371/journal.pgph.0002155 (PMC11356439; doi:10.1371/journal.pgph.0002155)
Supplement: Attachment — Submitted filename: Response to Reviewers May 2024.docx [file pgph.0002155.s013.docx]

Response to Reviewers

Re: PGPH-D-23-00926R1

7^th^ May 2024

Dear Editorial team,

We appreciate the time the editorial team and the reviewers have taken to peer review our manuscript entitled *"Insights into tuberculosis burden in Karachi, Pakistan: a concurrent adult tuberculosis prevalence and child M. tuberculosis infection survey"* which we think has strengthened the manuscript*.*

Based on the comments we have we have dropped Ultra trace positive culture-negative with a previous history of tuberculosis (n=4) from the case definition for the main analysis and have included a sensitivity analysis excluding all Ultra trace positive culture negative cases.

We have addressed the comments (in blue italics below) by both Reviewer #1 and the editor and provide a response highlighting where changes have occurred in the revised manuscript.

*Reviewer #1:  The authors have addressed the comments from the first review. The reviewer has only minor suggested corrections/comments.
Minor comments:
Introduction
- 2nd paragraph, line 4: Suggest delete “etc.” – not very scientific and not needed, as the examples start with “e.g.,”
- 2nd last line page 3: “examined” should be “examine”
Study design and setting
- line 4: “with had” should be “which had”
Child M. tuberculosis infection… (page 5)
- line 3: Suggest “…within the last…”
Page 7
- suggest sub-headings “Ethics approval” and “Role … source” to have similar font size/bold than “Statistical methods”
Table 1
- Suggest change “Known TB contact with last 2 years” to “Known TB contact within last 2 years”

Discussion
- Page 10, 3rd paragraph, line 1: Suggest use “…Xpert Ultra ‘trace’ only results…” (add “Xpert” and “results”)
- Page 10, 4th paragraph, last line: Should “the population” not be either “this population" or "the ‘prior ACF’ population?
- Page 11, 2nd paragraph, line 8: I think “then” at the end of the sentence should be deleted.*

We have made all the changes as suggested above and very much appreciate the time the reviewer has taken to improve the flow and readability of our manuscript.

*References
- Ref 3 – why all uppercase for author and title?*

I think that it is a quirk of the reference manager used but these have been manually edited to be in the same format as the other references

*- Ref 10 – Journal name abbreviation incorrect (double?)*

Yes this was indeed an error – many thanks for noticing. This has also been amended.

*- Ref 17 – URL and when accessed?*

URL has been added together with the date of last access.

*Editorial comments*

*Journal Requirements:

1. Please review your reference list to ensure that it is complete and correct. If you have cited papers that have been retracted, please include the rationale for doing so in the manuscript text, or remove these references and replace them with relevant current references. Any changes to the reference list should be mentioned in the rebuttal letter that accompanies your revised manuscript. If you need to cite a retracted article, indicate the article’s retracted status in the References list and also include a citation and full reference for the retraction notice.*

We have reviewed the reference list and are happy with the references.

*2. We do not publish any copyright or trademark symbols that usually accompany proprietary names, eg  ©, ®, ™  (e.g. next to drug or reagent names). Please remove all instances of trademark/copyright symbols throughout the text, including  ™ & ® on pages 4 &5.*

We have removed all instances of trademark/copyright symbols through the text.
